# Alterations of Tear Mediators in Patients with Keratoconus after Corneal Crosslinking Associate with Corneal Changes

**DOI:** 10.1371/journal.pone.0076333

**Published:** 2013-10-04

**Authors:** Bence Lajos Kolozsvári, András Berta, Goran Petrovski, Kata Miháltz, Péter Gogolák, Éva Rajnavölgyi, Ziad Hassan, Péter Széles, Mariann Fodor

**Affiliations:** 1 Department of Ophthalmology, Medical and Health Sciences Centre, University of Debrecen, Debrecen, Hungary; 2 Stem Cells and Eye Research Laboratory, Department of Biochemistry and Molecular Biology, Medical and Health Sciences Centre, University of Debrecen, Debrecen, Hungary; 3 Department of Ophthalmology, Faculty of Medicine, University of Szeged, Szeged, Hungary; 4 Department of Ophthalmology, Hietzing Hospital, Vienna, Austria; 5 Department of Immunology, Medical and Health Sciences Centre, University of Debrecen, Debrecen, Hungary; Cedars-Sinai Medical Center; UCLA School of Medicine, United States of America

## Abstract

Keratoconus (KC) is the most common primary corneal ectatic disease which has considerable importance in public health. Corneal collagen crosslinking (CXL) is a procedure to mitigate progression of KC and reduce demand for corneal transplantation. Although studies have proven the efficacy of CXL regarding corneal shape, none have investigated the effects of CXL on tear biomarkers which are useful tools to understand molecular mechanisms behind CXL. Our purpose was to determine the effect of CXL on tear mediators in patients with KC and analyze associations with corneal changes. Tear samples were collected pre-CXL from 26 eyes of 23 patients and during a 12-month follow-up. The mediators’ concentration was measured by Cytometric Bead Array technology. Corneal topography parameters measured by Scheimpflug Camera included: Thinnest-corneal-thickness (ThCT), keratometry values (K1, K2), Radii-Minimum (Rmin), Keratoconus-Index (KI), Center-KI (CKI), Index-of-Height Asymmetry (IHA) and Index-of-Surface Variance (ISV). At baseline, KI was correlated negatively with chemokine (C-C motif) ligand 5 (CCL5) (p=0.015) and matrix metalloproteinase (MMP)-13 (p=0.007). At day 4, interleukin (IL)-6 and IL-8 increased, while IL-13, IL-17A, interferon (IFN)-γ, CCL5, MMP-13, epidermal growth factor (EGF), nerve growth factor (NGF) and plasminogen activator inhibitor (PAI-1) decreased significantly compared to pre-CXL concentrations (p≤0.02). At 6 months tissue plasminogen activator (t-PA) increased (p=0.02), while at 12 months Rmin increased (p≤0.004), and IL-6 and CXCL8 (p=0.005 and p=0.047) as well as K1, ISV and KI decreased. After 6 months CKI and ISV showed significant associations with IL-17A; CKI with IL-13 and ThCT with IL-13 (p≤0.02), while at 12 months there were reverse associations between ThCT and IL-6, IL-13, INFγ, CCL5 and PAI-1 (p≤0.02). Alterations of mediators in tear fluid after CXL associate with topographic changes highlight the fact that many mediators are involved in the complex mechanisms after CXL. Further studies on biomarkers to investigate the efficacy of CXL are needed.

## Introduction

Keratoconus (KC) is a degenerative disorder characterized by progressive deformation of the corneal architecture including corneal thinning that gives rise to a cone shaped cornea [1-3]. Progression of KC is associated with an increase in the spherical component of the refraction and irregular astigmatism with consequent deterioration of visual acuity [1,2].

Corneal collagen crosslinking (CXL) is a procedure to mitigate the progression of KC [3-6]. Many clinical studies have provided data supporting the efficacy of the treatment: improvement in corneal shape, including a decrease in corneal higher-order aberrations, keratometry (K) values and several quantitative indices of corneal topography [5-10]. The findings did not always correlate with visual acuity or subjective visual symptoms [6,8,11].

Generally, KC is believed to be a non-inflammatory disease with multivariable origin [2]. Some studies have shown that degradation of stromal collagen is accompanied by expression of pro-inflammatory cytokines and matrix metalloproteinases (MMPs) and their inhibitors. Growth factors also play an important role in the pathogenesis [12-17]. In the tears of keratoconic patients increased interleukin (IL)-6, epidermal growth factor (EGF), MMP-1, -3, -7,-9, -13 and tissue inhibitor of metalloproteinases-1 (TIMP-1), and decreased interferon (IFN)-γ, IL-4, IL-5, IL-6, IL-8 (CXCL8), IL-12, IL-13, chemokine (C-C motif) ligand 5 (CCL5), vascular endothelial growth factor (VEGF) and alteration of tumor necrosis factor (TNF)-α and nerve growth factor (NGF) have been described as possible players in the disrupted corneal homeostasis [14-20]. The mediators determining the progression or stabilization of KC have not been well characterized and the effect of CXL on these factors is in its early state [16,17]. Although corneal biomechanical and architectural improvements after CXL have been well documented in the literature, to date, the changes in the tear film components have been yet explored. It is important to analyze whether these biomarkers may constitute determinant factors for the effectiveness of CXL.

In this study, to further asses the pathomechanism of KC after CXL, we evaluated the short and the long-term effect of CXL on the concentration of mediators (IL-6, -13, -17A, IFNγ, CXCL8, CCL5, MMP-9, -13, TIMP-1, tissue plasminogen activator (t-PA), plasminogen activator inhibitor (PAI-1), EGF and NGF in tears of patients with KC. In addition, the changes in concentration of mediators were correlated with the changes in outcomes: K values, Thinnest corneal thickness (ThCT), Radii Minimum (Rmin), Keratoconus-Index (KI), Center Keratoconus Index (CKI), Index of Height Asymmetry (IHA) and Index of Surface Variance (ISV)) measured by Pentacam over 12 months.

## Patients and Methods

Twenty-six eyes of 23 patients (mean age: 28.2 years, range: 16-60 years, standard deviation (SD): 10) with progressive KC were enrolled and treated in this prospective study. All eyes underwent comprehensive ophthalmological examination and tear sample collection before and after CXL during the 1-year follow-up period at regular intervals: preoperatively and at day 4, day 10 visits, and 1, 3, 6 and 12 months after CXL. Twelve eyes of 12 healthy controls (mean age: 27.8 years, range: 16-67 years, standard deviation (SD): 15.3) were also enrolled in this study.

Following the tenets of the Declaration of Helsinki, written informed consent was signed by all participants and/or their parent/guardian prior to enrollment. This study was approved by the Institutional Ethics Committee of the University of Debrecen. The inclusion criteria included 16 years of age or older and axial topography consistent with KC. Rabinowitz criteria were used for diagnosing KC [21]. Progressive KC was defined as 1 or more of the following changes over 24 months: an increase of 1.0 diopter (D) or more in the steepest K value, an increase of 1.0 D or more in manifest cylinder, or an increase of 0.5 D or more in manifest refraction spherical equivalent [6,11]. Exclusion criteria were: previous ocular surgery, abnormality in lens or retina on biomicroscopic examination, chemical injury or delayed epithelial healing, corneal pachymetry less than 300 μm, and pregnancy or lactation during the course of the study.

### Crosslinking Treatment

CXL was performed using InPro CCL-Lix device (Norderstadt, Germany). Topical anesthesia was administered and the corneal epithelium removed by mechanical debridement over the central 8.0 mm. A 0.1% riboflavin solution (with 20% dextran) was then administered topically every 2 minutes for 30 minutes. After riboflavin administration, its absorption throughout the corneal stroma and anterior chamber was confirmed by a slitlamp examination. Pachymetry (obtained with Pentacam) was performed and if the cornea was less than 400 μm (4 out of 26 eyes), hypotonic riboflavin (0.1% in sterile water; Medio Cross sine, Medio-Haus Medizinprodukte GmbH) was administered, 1 drop every 10 seconds for 2 minute sessions, after which pachymetry was performed to confirm that the stroma had swollen to 400 μm or more [11,22]. This was repeated until adequate corneal thickness was obtained. The cornea was exposed to ultraviolet-A (UV-A) 365 nm light for 30 minutes at an irradiance of 3 mW/cm^2^. The postoperative treatment was antibiotic eye drops for 7 days (tobramycin), steroid eye drops (fluorometholone) and artificial tear drops for at least 3 months. No contact lenses were used postoperatively.

### Measurements

All eyes had a complete ophthalmological evaluation, including keratometry, best-corrected visual acuity measurements, slit-lamp biomicroscopy (under low illumination to avoid reflex tearing), and Rotating Scheimpflug topography (Pentacam HR, Oculus Optikgeräte GmbH, Wetzlar, Germany) before CXL and during the 1-year follow-up, at each opthalmological visit. The following data were exported to Microsoft Excel (Microsoft Corp, Redmond, Washington): Holladay equivalent keratometry values in the flat (K1) and steep (K2) meridian, ThCT, Rmin, KI, CKI, IHA, ISV. For height data measurements, the toric ellipsoid reference surface was used [10]. The stage of KC was graded as mild when the steepest keratometric reading (K2) was <45 diopters (D), moderate if K2 was between 45 and 52 D, and severe if K2 >52 D. K2 has been considered to be a reliable quantitative clinical variable to assess the severity of KC [13,16].

### Tear collection and analysis

Non-stimulated tear samples were collected from all 26 eyes of 23 keratoconic patients before CXL and during the 1-year follow-up at each opthalmological visit and once from the 12 control eyes. The tear collection was carried out with capillary tubes from the inferior meniscus, without topical anesthesia for 2 min and the total volume of the collected tears registered [23]. The samples were immediately transferred to Eppendorf tubes and frozen at -80 °C without centrifugation within 15 min from collection. Usually, 4 µl tear samples or more could be collected. In keratoconic patients, the concentrations of IL-6, CXCL8 (IL-8), CCL5 (RANTES), MMP-9, MMP-13, TIMP-1, NGF, tPA and PAI were measured in all 157 tear samples, while the volume of 105 tear samples allowed a second measurement for IL-13, IL-17A, INFγ and EGF by the Cytometric Bead Array method. Combined FlowCytomix™ Simplex Kits were used with the appropriate FlowCytomix Basic Kit with minor modifications of the manufacturer’s instructions (eBioscience, Bender MedSystems GmbH, Vienna, Austria). Briefly, 12.5 µl of tear samples (if needed, diluted samples) or serial dilutions of mixed standard cytokines were added to 12.5 µl suspension of fluorescent cytokine capture beads in multiwell filter microplates. 12.5 µl of biotin conjugated anti-cytokine antibody was added to the wells, then the plates were incubated for 2 hours on a microplate shaker. The wells were emptied and washed with a vacuum filtration manifold. Phycoerythrin conjugated streptavidin was added to the wells followed by additional incubation for 1 hour and washing. 150 µl assay buffer was applied to the wells, then multiparametric data acquisition was performed on a FACS Array cytometer (BD Biosciences Immunocytometry Systems, San Jose, CA). Data were analyzed with the FlowCytomix Pro 2.3 software. Additional serial dilutions of the standard were applied to obtain better sensitivity and therefore, modified standard curves were generated in the analysis. The detection limits were the following: IL-6 (1.2 pg/ml), IL-13 (4.5 pg/ml), IL-17A (2.5 pg/ml), INFγ (1.6 pg/ml), CXCL8 (0.5 pg/ml), CCL5 (25 pg/ml), MMP-9 (95 pg/ml), MMP-13 (50 pg/ml), TIMP-1 (28 pg/ml), NGF (126.8 pg/ml), EGF (22.7 pg/ml), tPA (4.8 pg/ml) and PAI-1 (13.5 pg/ml).

### Statistical methods

Variables were described in terms of means and SD on their native scales. For all analytical procedures, keratometric variables and mediators in the tear samples were transformed to improve normality using zero-skewness log transformation.

Paired 2-tailed Student’s t tests were used to analyze post-CXL changes from baseline for all 13 mediators and the 8 variables obtained from Pentacam.

Multilevel mixed-effects linear regression was used to analyze the association between the concentration of the 13 mediators and keratometric readings obtained from Scheimpflug Camera (ThCT, K1, K2, Rmin, KI, CKI, IHA, ISV). Models were fitted separately for each possible pairing between mediators and keratometric variables, with adjustment for contact lens wear and tear volume, and interaction terms between measurement occasion and mediator concentration. The statistical package applied was Stata version 11. The significance criterion was set at α=0.05.

## Results

Twenty-six eyes of 23 keratoconic patients were enrolled and underwent CXL in this study (mean age: 28.2+10 years). The KC was classified as follows: grade 1, 2 eyes (8%); grade 2, 12 eyes (46%); grade 3, 12 eyes (46%). The mean follow-up was 11 months (157-494 days), 18 eyes were followed up to one year post-CXL.

**Figure 1:**
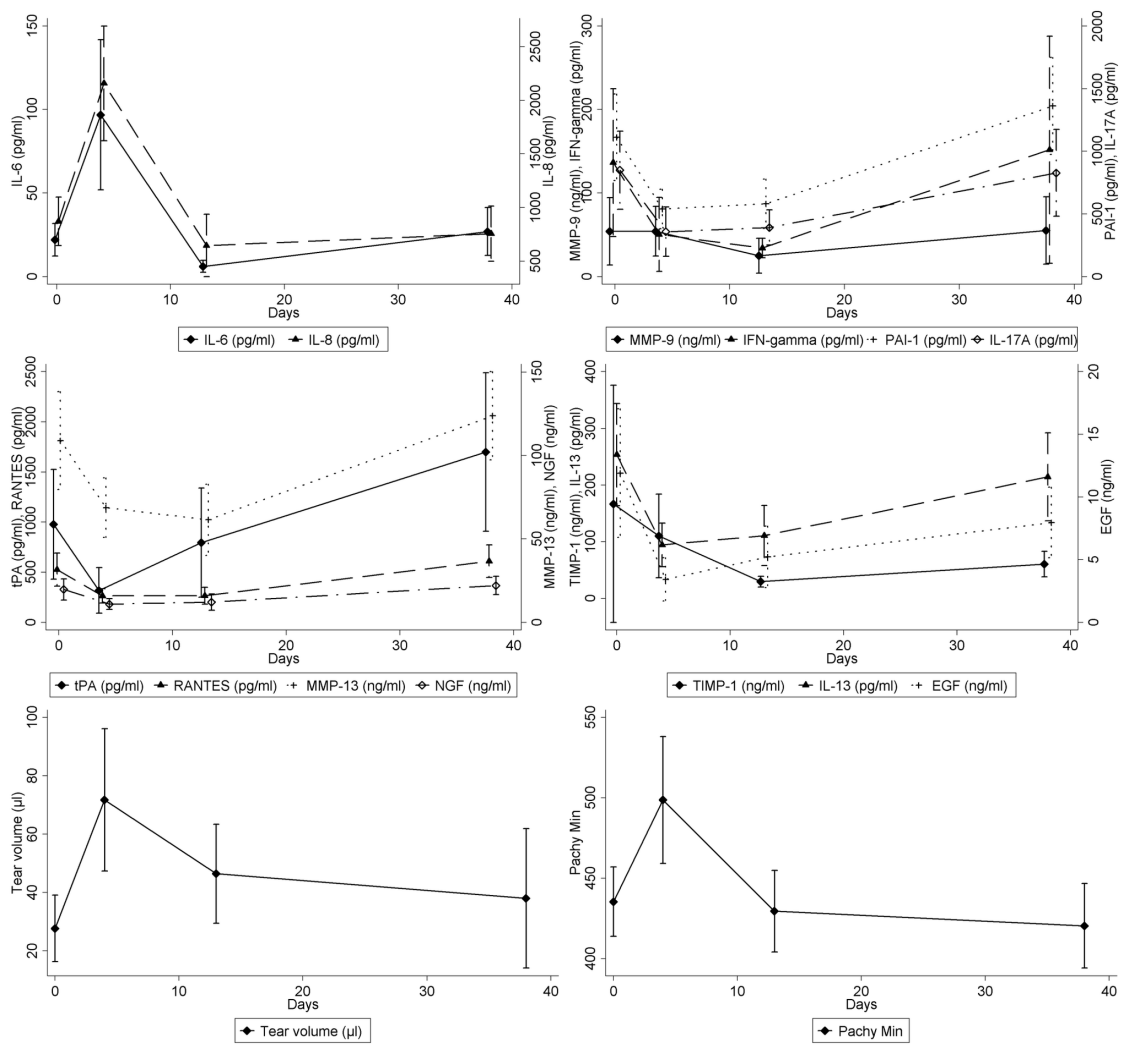
Short-term changes in the concentration of mediators in the tear fluid after corneal collagen crosslinking. **a.** the concentration of interleukin(IL)-6 and IL-8 (CXCL8) increased 4 days after CXL; **b.** matrix metalloproteinase(MMP)-9, plasminogen activator inhibitor (PAI)-1, IL-17A and IFN-gamma showed no changes 38 days after CXL compared to the baseline; **c.** tissue plasminogen activator (tPA), RANTES (CCL5), MMP-13 and nerve growth factor (NGF) increased slightly 38 days after CXL compared to the baseline; **d.** tissue inhibitor of metalloproteinases (TIMP)-1, IL-13 and epidermal growth factor (EGF) decreased slightly 38 days after CXL compared to baseline; **e-f.** Short-term changes in the collected tear volume and the thinnest corneal thickness (Pachy min.).

Short-term non-transformed data were collected up to 38 days from CXL (Figure 1). As expected, the collected tear volume (p<0.0001) and the thinnest corneal thickness (p=0.0005) increased significantly 4 days after the treatment. Despite the excessive tearing at day 4, there were statistically significant increases in the concentrations of IL-6 and CXCL8 (p<0.0001) when compared to the pre-operative (pre-CXL) baseline levels. At the same time, the concentration of IL-13 (p=0.01), IL-17A (p=0.001), IFNγ (p=0.02), CCL5 (p=0.001), MMP-13 (p=0.02), EGF (p<0.0001), NGF (p=0.01) and PAI-1 (p=0.001) decreased significantly, and there were no significant changes in the concentrations of MMP-9, TIMP-1 and t-PA. The changes in the concentration of all the mediators 38 days after CXL compared with the baseline failed to reach statistical significance, and the volume of the collected tears returned back to the pre-CXL levels. The thinnest corneal thickness were significantly decreased (p<0.0001) after CXL compared to the baseline data at day 38.

The pre-CXL and post-operative long-term data in patients with keratoconus and the data from the healthy controls, including the Pentacam results and the concentration of the mediators without any adjustment are shown in Table 1 (significant differences at 6 and 12 months compared to the baseline data with adjustment are indicated). At 6 months, there was a significant decrease in ThCT and ISV (p=0.03 and p=0.0005) and increase in Rmin (p=0.007). At 12 months, the change in K1, ISV and KI were statistically decreased compared to the baseline (p=0.0045, p=0.002, p=0.0005; respectively), while Rmin was increased (p=0.0004). At 6 months, there was a significant increase in tPA (p=0.023), while at 12 months, there was a significant decrease in IL-6 and CXCL8 (p=0.005 and p=0.047). The changes in other enzymes and growth factors compared to the baseline failed to reach statistical significance at 6 months or 12 months. However, when the pre-CXL levels of the mediators were divided into low or high subgroups and were analized individually, many significant changes were observed at the two observed time points. In the low concentration pre-CXL subgroup, a significant elevation in IL-6 (p=0.016), IL-13 (p=0.001), IL-17A (p=0.004), IFNγ (p=0.013), CCL5 (p=0.013), MMP-9 (p=0.01), MMP-13 (p=0.0003), TIMP-1(p=0.011), NGF (p=0.04), tPA (p=0.007) and PAI-1 (p=0.017) was observed at 6 months, while only the IL-13 (p=0.009) and MMP-13 (p=0.024) were elevated at 12 months. In the high concentration pre-CXL subgroup, significant decreases were observed after 6 months in IL-13 (p=0.007) and IL-17A (p=0.001), while at 12 months the IL-6 (p=0.0002), IL-13 (p=0.049), IL-17A (p=0.019), CXCL8 (p=0.003), CCL5 (p=0.0012), MMP-9 (p=0.025), MMP-13 (p=0.047), NGF (p=0.014), EGF (p=0.013) and PAI-1 (p=0.007) showed the same decreasing trend as IL-13 and IL-17A after 6 months. The concentration of all the examined mediators in the tear samples from the control eyes were significantly lower when compared to the pre-operative (pre-CXL) baseline levels in patients (p<0.05).

**Table 1:** Mean (SD) values of the measured parameters over a 12 months follow-up period in patients with keratoconus and in healthy controls.

**Parameter**	***K1 (D)***	***K2 (D)***	***Rmin (mm)***	***ThCT (μm)***	***ISV***	***IHA***	***KI***	***CKI***	***IL-6 (pg/ml)***	***IL-13 (pg/ml)***	***IL-17A (pg/ml)***	***INFγ (pg/ml)***	***CCL5 (pg/ml)***	***CXCL8 (pg/ml)***	***MMP-9 (ng/ml)***	***MMP-13 (ng/ml)***	***TIMP-1 (ng/ml)***	***tPA (pg/ml)***	***PAI-1 (ng/ml)***	***EGF (ng/ml)***	***NGF (ng/ml)***
**PreCXL**	47.0 (4.4)	52.1 (5.4)	5.69 (0.6)	435.4 (55.9)	116.3 (44.1)	42.0 (23.8)	1.31 (0.13)	1.08 (0.07)	22.0 (25.3)	253.6 (205.4)	849.2 (710.2)	136.5 (201.9)	525.7 (427.5)	871 (583)	54.2 (105.1)	108.9 (76.3)	166.9 (543.8)	977 (1425)	1.11 (0.89)	11.91 (11.7)	19.7 (16.6)
**PostCXL-6 months**	47.3 (4.6)	52.4 (5.4)	*5.74 (0.6)	*424.7 (57.4)	*112.6 (47.4)	38.6 (22.1)	1.31 (0.14)	1.31 (0.14)	30.0 (35.8)	212.0 (103.5)	850.7 (555.3)	122.2 (117.5)	620.2 (506.5)	1089 (1155)	67.3 (119.8)	133.2 (80.6)	203.8 (509.0)	*1843 (2157)	1.38 (1.11)	7.95 (5.9)	23.3 (18.7)
**PostCXL-12 months**	**46.7 (3.8)	51.7 (4.9)	**5.83 (0.7)	444.2 (61.8)	**107.9 (59.9)	36.4 (28.0)	**1.29 (0.13)	1.29 (0.14)	**14.9 (28.8)	249.9 (327.2)	872.6 (864.8)	204.1 (424.5)	455.1 (416.2)	**688 (394)	50.0 (96.8)	108.6 (69.0)	85.6 (163.5)	729 (1314)	1.08 (0.93)	7.70 (7.4)	17.1 (13.6)
**Controls**	43.5 (1.2)	44.5 (1.4)	7.51 (0.3)	558.3 (39.8)	19.7 (5.6)	6.0 (3.6)	1.03 (0.02)	1.01 (0.0)	2.4 (3.7)	93.1 (158.1)	167.3 (307.4)	25.1 (39.6)	145.5 (193.5)	468.5 (287.6)	20.7 (24.5)	14.9 (20.1)	22.0 (19.5)	379 (99)	0.59 (0.60)	3.03 (3.6)	8.0 (2.8)

Pentacam results: K1, K2=Holladay equivalent keratometry values; Rmin=Radii Minimum; ThCT=Thinnest corneal thickness; ISV= Index of Surface Variance ; IHA= Index of Height Asymmetry; KI=Keratoconus-Index; CKI=Center Keratoconus Index; Mediators measured in the tear samples: IL=interleukin; CXCL8=IL-8; CCL5=RANTES; MMP=matrix metalloproteinase; TIMP=tissue inhibitor of metalloproteinases; tPA=tissue plasminogen activator; PAI=plasminogen activator inhibitor; NGF=nerve growth factor; EGF=epidermal growth factor.

* p<0.05 change between pre-CXL (baseline) and 0.5 year in patients; **= p<0.05 change between pre-CXL (baseline) and 1 year in patients

At baseline, KI was correlated negatively with CCL5 (p=0.015) and MMP-13 (p=0.007). We then examined the linear association between the different mediators and the tomography data. After 3 months, KI was associated negatively with IL-17A (p=0.002) and MMP-13 (p=0.01); similarly IHA was negatively associated with IL-17A (p=0.016) and EGF (p=0.032). After 6 months, CKI and ISV showed significant associations with IL-17A (p=0.001 and p=0.016, respectively), similar to CKI with IL-13 (p=0.002) and ThCT with IL-13 (p=0.01). After 12 months, there were reverse associations between the thinnest corneal thickness (ThCT, Pachy Min) and IL-6 (p=0.005), IL-13 (p= 0.017), IL-17A (p=0.049), INFγ (p=0.017), CCL5 (p=0.02) and PAI-1 (p=0.011).

## Discussion

To the best of our knowledge, there are no studies evaluating the effect of the corneal cross-linking procedure on different tear biomarkers. Our results show significant changes in the level of several proteins including cytokines, chemokines, enzymes (and inhibitors) and grow factors in tear samples after CXL with concomitant changes in the shape of KC corneas. Although this morphological regression effect of CXL has already been reported by several previous studies [3,5-11,24,25], this is the first high-throughput study to demonstrate the effect of CXL on tear mediators. In this study, the postoperative changes in 8 Pentacam topography indices were evaluated and the associations between the different protein concentrations were followed up to 12 months providing a more comprehensive analysis of the potential effect of CXL on keratoconic corneas. The only study investigating the expression of mediators after CXL has involved patients 3 to 6 months postoperatively, and tears were collected during single study visits without comparing the data with the pre-CXL baseline-levels [17]. Our study collected tear samples from patients with progressive KC and at regular intervals during 1 year after CXL. Cytokines, chemokines, enzymes, and growth factors are important in wound healing and tissue redistribution response of corneal tissue, regulating the wound healing, apoptosis, cell cycling and migration processes under physiological or pathological conditions. It is assumed that biomarkers examined in our study and others from this complex network may be responsible for clinical outcomes after CXL treatment.

Previous studies of patients with progressive KC after CXL found significant improvement in several Pentacam topography indices (CKI, KI, IHA, Rmin, ISV and IVA) [5-10,24]. The improvements of K1, KI, ISV, ThCT and Rmin detected in our study are consistent with these previous findings supporting the fact that the cornea is assuming a more regular shape after CXL. Our results are in line with the previous findings that collagen CXL is an effective procedure.

The CXL treatment of de-epithelialized corneas causes a prompt, excessive release of several mediators independent of the long-term effect of the procedure, and due to the mechanical (epithelial removal), chemical (riboflavin soaking) and physical (UVA irradiation) stress on the cornea. Since the volume of the collected tear volumes during 2 mins can differ significantly in the post-CXL period at 1 month compared to the pre-CXL volumes, and due to the diluting effect from excessive tearing on the ocular surface, the estimates of the mediator concentrations on keratometric readings were adjusted for the collected tear volume. Besides the presence of excessive tearing under certain conditions, the concentration of IL-6 and CXCL8 significantly increased in our study 4 days after CXL (p<0.0001), suggesting the prompt involvement of inflammatory cells in the process. By day 4, the ThCT increased significantly (p=0.0005) and this swelling dramatically decreased at day 38 (p<0.0001). This edematous process in the corneal stroma in the early post-CXL phases has been already described before [26]. The concentration of several mediators decreased significantly because of the diluting effect of the tearing. The CXL treatment retards the progression of KC by cross-linking of collagen molecules. Although the complex pathophysiology of CXL is unclear, the early clinical worsening and the transitional alteration of the mediators coincide with the epithelial debridement, re-epithelization process and post-operative keratocyte apoptosis and repopulation as well as new collagen synthesis [8,26,27]. UVA causes the keratocytes in the outer layers of the treated part of the cornea to undergo cell death [7]. By definition, in order to crosslink tissue, keratocytes must be killed. When cells die through necrosis, a strong inflammatory response is initiated and a release of various mediators including chemokines occurs. IL-1 is the master regulator of sterile inflammation and is derived from necrotic corneal epithelial cells which can induce the production of IL-6, stimulating the migration of corneal epithelial cells [28]. Necrotic corneal epithelial cells enhance the production and the release of CXCL8, and with its chemotactic activity CXCL8 can influence the inflammatory cells. In the early-phase after CXL, the extremely elevated release of IL-6 and CXCL8, detected in our study, contribute significantly to the corneal epithelial wound healing process and the recruitment of inflammatory cells into the corneal stroma, resulting in prevention of tissue damage from excessive inflammation by clearance of damaged cells [28]. After CXL, all corneal layers regenerate rapidly, even the epithelial regrowth is complete after four days [27]. After re-epithelisation of the cornea, remodelling and reorganization occur, new keratocytes migrate into the central area over several months following CXL. In the early stages of the recovery from CXL and at 38 days after CXL, the changes in the concentration of all the mediators and even the volume of the collected tears during 2-min return back to the pre-CXL levels. Gradual repopulation of the corneal stroma, starting between the second and third month after the intervention, is usually completed within six months [27].

KC is definied as a non-inflammatory disease of the cornea, but increasing number of studies show an over-expression of several cytokines in it [16,17], therefore, classifying the disease as non-inflammatory may be inappropriate [29]. The decrease in the concentration of IL-6 (p=0.005) 12 months after CXL shown in the current study supports the previous reported observation that IL-6 is the only cytokine which significantly differed in the tears of KC subjects compared to the CXL group [17]. In addition, we have shown a strong negative correlation between IL-6 and ThCT 1 year after CXL. We could detect significant decrease of CXCL8 at that time point, which also supports the assumption that cytokines and chemokines play an important role in the pathomechanism of KC and that CXL treatment may be able to alter the inflammatory response. CXCL8 is a potent chemotactic factor in the human tears. A decreasing trend towards the T_H_1 cytokines (IFNγ) in KC, may suggest presence of complex imbalance in the cytokines leading to altered epithelial and stromal function in this disease [16]. The decrease in the levels of IL-6 and CXCL8 after CXL treatment could be considered as positive for corneal health because the levels of these cytokines of healthy controls in our study were significantly lower compared to the patients. This might be a contributing factor in the stabilization of this corneal disease. The analysis of the long-term associations after CXL have revealed reverse association between ThCT and IFNγ. Jun et al. observed decreased IL-13 level in tears of severe KC and an increasing trend in IL-17 [16]. IL-13 plays crucial roles in amplification of the T_H_2 response, and the decreased levels suggest that T_H_2 responses may be dampened in KC [16]. Dermal fibroblasts stimulated with IL-13 upregulate the production of collagens type I and III [30]. IL-17 is produced primarily by the T_H_17 subset T lymphocytes, and it can also mediate induction of fibroblasts as well as production of tissue degrading proteases and cytokines [31]. IL-13 and IL-17 in our study showed significant long-term (6 and 12 months) associations with topography indices (ISV, CKI, ThCT). Additional studies are needed to further validate the role of IL-13 and IL-17 in tissue damage and the effect of CXL in KC. Our baseline findings that KI correlates negatively with CCL5 is in line with Jun et al. - the only group having reported tear sample measurements of CCL5 and reporting lower levels in KC [16]. Our baseline observation and a reversely significant association between ThCT and CCL5, 1 year after CXL underline the importance of CCL5 in the pathomechanism of KC.

MMPs are secreted in response to cytokines and growth factors and elevated levels of MMPs in the tear fluid of KC patients indicates a tissue degenerative process contributing to the thinning of the cornea [14,17,19]. MMPs and cytokines interact with each other forming a complex network, including the stimulation of MMP-9 and MMP-13 by IL-6 [17]. The active form of t-PA converts plasminogen to plasmin, which can also degrade several components of the extracellular matrix and trigger activation of the MMP pathway. MMPs and PAs in turn are partially regulated by TIMPs and PAIs, inhibiting this cascade system and therefore influencing KC progression. The PAI-1 gene can be induced by several growth factors and cytokines, and it can inhibit the activity of t-PA enzymes. A significant increase in tPA was detected in our study 6 months after the CXL treatment and after 1 year, a reverse association was observed between ThCT and PAI-1. MMPs participate in extracellular matrix remodelling following CXL, and a positive correlation between keratometry and MMP-13 and TIMP-1 levels in the tear film in KC has been observed by others without any information on the pachometric data [17]. In our study, a negative correlation could be observed between concentrations of MMP-13 and KI at baseline and 3 months post-CXL. TIMPs are natural inhibitors of the different MMPs, and there have been various studies with conflicting reports on the expression of TIMP-1 in KC corneas [15,17,32,33]. In our study, we could not detect any correlation or alteration in the levels of MMP-9 and TIMP-1, which suggests, that other MMPs, as well as other enzymes might play a more crucial role in the underlying molecular mechanism following CXL, and probably, the actual enzyme activities influence the final effect. TIMP-1 has been observed to prevent TIMP-3 induced apoptosis of keratocytes [33], therefore the lack of the alteration of TIMP-1 concentration after CXL may be beneficial to KC patients. Since protease activity has not been analized in this study and it may have importance in the pathogenesis of KC (significantly elevated activity of collagenases have been found, while reduced protease activities have been observed after CXL) [17,34,35], future studies may need to confirm the active interplay between MMPs and ILs.

Early post-operative *in vivo* confocal microscopic analysis has shown an immediate disappearance of the subepithelial plexus and the anterior-midstromal nerve fibers [25]. The nerve regeneration after CXL is very rapid, and is almost complete six months after the treatment [27]. The ocular surface healing, including corneal epithelial cell proliferation and immune-modulating actions of NGF have been extensively studied [36]. In our study, during the early postoperative period, when the re-epithelization process is dominant, a slightly increase in NGF could be observed, but no association or long-term changes in the concentration of NGF could be detected after CXL. The role of NGF in the pathogenesis of KC is under investigation and because its role in the proliferation of limbal epithelial progenitor cells is suggested [36], further studies are required to demonstrate the importance of NGF after CXL treatment. EGF is produced by lacrimal glands and plays a role in cell proliferation, migration and apoptosis as well, and has been found to be up-regulated after epithelial injury [37].

It is important to mention here that the use of steroid eye drops and their duration after CXL highly depend on the ophthalmologists’ practice. In generally, steroids are administered 0-4 weeks after CXL treatment [5-8,11,22,27]. In contrast, we have used fluorometholone drops for minimum 3 months and found no increase in the mean intraocular pressure postoperatively (data not shown). Corneal thickness and posterior elevation at minimum pachymetry proved to be highly reliable diagnostic parameters of KC and to monitor the treatment efficacy after CXL [10]. Currently, the long-term effects of CXL treatment are not well known [4,7], but based on our findings, the steroid treatment may also influence the post-CXL changes. The causes of the increase in central corneal thickness after the second year described by Raiskup-Wolf et al. and our findings that ThCT did not decrease 1 year after CXL should be studied further [4]. The standardized, applied CXL protocol supposes approximately equal penetration depth in all eyes - the CXL may affect the deeper stromal tissues of thinner corneas compared to less advanced cases with thicker corneas [10]. Since steroids have been shown to inhibit collagen synthesis, prevent collagen accumulation and decrease collagen turnover in animal models, steroid therapy has been recommended to be prolonged over a month only in case of risk of corneal opacity after CXL [27].

To better ascertain differences in response to CXL, low and high baseline levels of mediators subgroups were compared here. Many significant long-term changes have been observed in our study and the different trends may indicate the different effects mediators in the tear film may have, playing important role in the cornea homeostasis.

A limitation of this study is that it cannot exclude the possibility of other mediators being involved in the post-CXL period and the identification of the source and activity of the mediators and the expression of the different receptors. Furthermore, we could compare the pre- and the post-CXL findings with the baseline data of the patients and also with a healthy control group, but ideally, because of possible progression of the disease that can be accompanied with alteration in the mediator levels, the same number of fellow eyes with the same severity of KC without any treatment would have been desired to be the control group, but when progression is detected, treatment is usually indicated and the long term results would be limited to reach. Although it has been demonstrated that mediators are associated with the severity of KC, it is unclear which proteins can be used to distinguish non-progressive form of KC from progressive, where the early CXL treatment would be very important. It would be of great benefit to understand which biomarkers may promote the effect of CXL treatment. Tear samples are an essential tool to understand the molecular mechanism behind CXL and the multiplex platform is ideally suited for the detection of biomarkers from tear samples. Several other types of mediators would be interesting to measure, but without pooling the tear samples, the number of the proteins that can be measured is limited. Despite these limitations, it is important to emphasize that our results highlight the fact that many mediators are involved in the complex mechanisms after CXL. It remains to be determined in further studies which of these mediators or any others are critical in the etiopathogenesis of the disease and have significance after CXL.

In summary, this study reveals many mediators being altered in the tears of KC patients after CXL, and these alterations may have an impact on the effect of this treatment. As a next step, the precise role of these mediators needs to be defined and it is important to confirm the observed changes in a larger study to gain further insight into the molecular alterations after corneal CXL treatment. This might then serve as a platform for local inhibition of pathologic corneal thinning or individualized treatment. Further studies determining the pre-operative predictors of patients in whom outcomes significantly improve or worsen after CXL treatment are also needed. Additionally, studies are required to further elucidate the effect of the change of mediators after CXL with the different stages of KC.
